# Learning to echolocate in sighted people: a correlational study on attention, working memory and spatial abilities

**DOI:** 10.1007/s00221-016-4833-z

**Published:** 2016-11-25

**Authors:** M. R. Ekkel, R. van Lier, B. Steenbergen

**Affiliations:** 1 Behavioural Science Institute, Radboud University Nijmegen, Postbus 9104, 6500 HE Nijmegen, The Netherlands; 2 Donders Institute for Brain, Cognition and Behaviour, Radboud University Nijmegen, Nijmegen, The Netherlands; 3 School of Psychology, Australian Catholic University, Melbourne, Australia

**Keywords:** Echolocation, Visual impairment, Perception, Attention

## Abstract

Echolocation can be beneficial for the orientation and mobility of visually impaired people. Research has shown considerable individual differences for acquiring this skill. However, individual characteristics that affect the learning of echolocation are largely unknown. In the present study, we examined individual factors that are likely to affect learning to echolocate: sustained and divided attention, working memory, and spatial abilities. To that aim, sighted participants with normal hearing performed an echolocation task that was adapted from a previously reported size-discrimination task. In line with existing studies, we found large individual differences in echolocation ability. We also found indications that participants were able to improve their echolocation ability. Furthermore, we found a significant positive correlation between improvement in echolocation and sustained and divided attention, as measured in the PASAT. No significant correlations were found with our tests regarding working memory and spatial abilities. These findings may have implications for the development of guidelines for training echolocation that are tailored to the individual with a visual impairment.

## Introduction

Human echolocation is the ability to detect sound reflecting surfaces by sound echoes (for a review see Kolarik et al. 2014). Visually impaired people can use this ability to detect objects and to determine the distance to an object (Kellogg 1962; Rice et al. 1965; Schenkman and Nilsson 2010; Supa et al. 1944). Research furthermore indicated that humans can learn to determine the size, shape and even texture of an object using echolocation (Arnott et al. 2013; Hausfeld et al. 1982; Kellogg 1962; Milne et al. 2014a; Rice 1967; Rice and Feinstein 1965; Teng and Whitney 2011; Thaler et al. 2014). As such, echolocation can provide real-life advantages in orientation and mobility for blind people and may be additive to the information that they gain from other sources such as the white cane. In a recent survey study, Thaler (2013) found a positive correlation between the use of echolocation and mobility in unfamiliar places, indicating that echolocation may be of additive value for blind people. More recently, it was shown that the use of echolocation is not only useful for navigating, but can also have important benefits for the representation of auditory space in general (Vercillo et al. 2015).

At present, no systematic programs to train echolocation skills are available in the literature (Ekkel et al. 2014; Kolarik et al. 2014). Echolocation is predominantly trained by orientation and mobility instructors at visual rehabilitation centres, but systematic guidelines or protocols have not yet been developed. This lack of specific protocols is likely to be due to the large individual differences in the extent to which visually impaired people can learn echolocation. In line with this, it was reported that some individuals reach expert levels following training while others still remain at a lower level (Kolarik et al. 2014; Worchel et al. 1950).

To advance insights into the learning of echolocation, the next step is to disentangle the mediating individual factors affecting the learning of this skill. Teng et al. (2012) observed a correlation between the age of onset of blindness and echolocation performance. Individuals that became visually impaired earlier in life generally perform better at echolocation tasks than people that became visually impaired later in their life. Factors that may contribute to this finding are the greater experience with auditory information, resulting from a longer period of dependence on non-visual stimuli in daily life and cross-modal plasticity, resulting from visual deprivation early in life (Heimler et al. 2014; Voss 2013). Other factors that may contribute to individual differences in the learning of echolocation are largely unknown. Already in 1967, Rice suggested that the individual differences in performance that cannot be attributed to differences in the signal or environment may be attributed to variations in attention, motivation, intelligence, or personality (Rice 1967). Despite this early suggestion, only one recent study has addressed the question as to what non-auditory aspects of cognition might underlie individual differences in echolocation ability (Thaler et al. 2014). In this study, it was found that echolocation ability was positively correlated with vividness of visual imagery in sighted participants (Thaler et al. 2014). More research on the relation between echolocation ability and cognitive factors is warranted, such that individual differences can be taken into account when designing tailor-made echolocation-learning protocols. Consequently, efficacy of the training will be advanced.

In auditory perception in general, the relevance of cognitive variables has been repeatedly acknowledged (Zekveld et al. 2013). Even the most basic auditory tasks are affected by top-down processing, because auditory skills are closely related to executive processes, including working memory and attention (Moossavi et al. 2014). Working memory is the system that is responsible for the holding and processing of new and already stored information. It is the capacity to perform task-relevant processing of information kept in mind (Baddeley and Hitch 1974). In sound localization in particular, evidence was found for the involvement of top-down processing (Merat and Groeger 2003). Using a dual-task paradigm, Merat and Groeger (2003) found that performance in sound localization was not impaired when participants had to repeat single digits or digit spans concurrently. However, performance in sound localization was impaired when the Paced Visual Serial Addition Test, a task for working memory and attention, was performed concurrently. This suggests the influence of executive processes in sound localization. Extending this reasoning, it may be hypothesized that also in echolocation, top-down processing is involved. Further evidence suggesting the role of top-down processing in echolocation stems from research concerning the precedence effect, an echo suppression mechanism (Litovsky et al. 1999; Brown et al. 2015; Wallach et al. 1949). It was found that the precedence effect was reduced during an active echolocation task in comparison with a passive listening task (Wallmeier et al. 2013), suggesting the involvement of central cognitive processes in echolocation. In the current study, we tested whether individual differences in working memory, sustained attention, and divided attention are related to the learning of echolocation.

Furthermore, we explored the role of spatial cognition in the learning of echolocation. Spatial cognition relates to the way in which humans perceive, mentally represent, and interact with the spatial characteristics of their environment. These characteristics include object and scene properties, such as size, shape, and scale, as well as relations among objects, such as distance, direction, orientation, and location (Waller and Nadel 2013). As echolocation is also closely related to determining the spatial characteristics of the environment, we scrutinized the possible role of spatial cognition for learning to echolocate.

In sum, the aim of the present study was to examine individual factors that may contribute to the learning of echolocation. Echolocation ability is assessed by using an adaptation of a previously used size-discrimination task (Teng and Whitney 2011; Thaler et al. 2014), in which participants have to judge the relative positions of two differently sized discs, by using echolocation. Additionally, the role of spatial abilities, working memory, sustained attention, and divided attention in echolocation ability is researched.

## Materials and methods

All testing procedures were approved by the Ethics Board (ECSW2013-1811-150) of the Faculty of Social Sciences at the Radboud University. Participants gave written informed consent prior to testing.

### Participants

Twenty-three sighted participants were recruited for this study. Participants were undergraduate students at Radboud University and were compensated with course credits. Of the 23 participants, 5 participants did not move their head at all during the echolocation task. As shown in previous research, echolocation experts who were not allowed to move their head during echolocation performed at chance level when determining the shape of an object (Milne et al. 2014b). In our experiment, performance of the five participants that did not move their head did not differ from chance level. This shows that head movements are essential for proper echolocation. Therefore, these five participants were excluded from further analyses, yielding the results of 18 participants (four males), aged between 18 and 24 years (*M* = 19.50; SD = 1.72). These 18 participants moved their head horizontally, vertically, or both, during trials. We performed pure-tone hearing audiometry in the 250–4000 Hz range to establish that all participants had normal hearing (<20 dB hearing loss).

### Stimuli and procedure

The experiment took place on four separate days. On day one and two, participants completed the echolocation task, consisting of two sessions per day. The echolocation task took between 1 and 2 h per day to complete. On day three, participants completed the spatial abilities task, digit span, and the Paced Auditory Serial Attention Task (PASAT). These were completed between 1 and 2 h. On day four, participants’ hearing ability was assessed.

#### Echolocation task

**Fig. 1:**
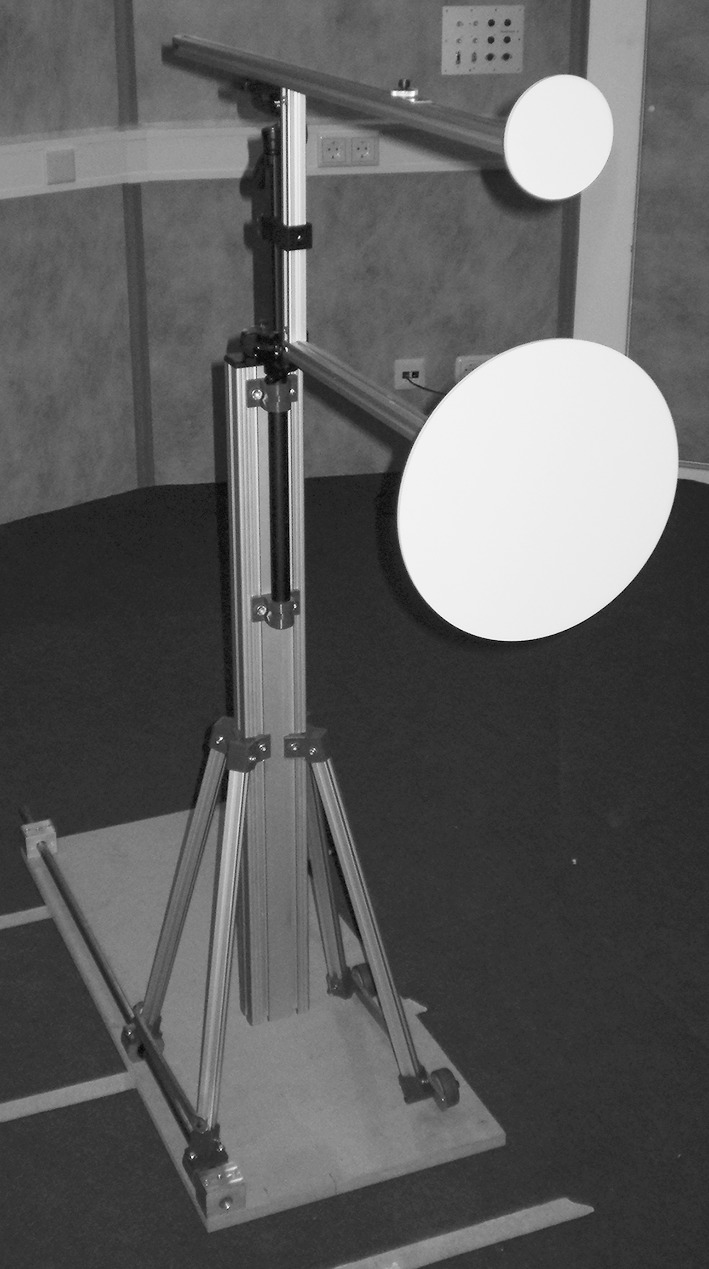
Set-up of the echolocation task

The experiment took place inside a soundproof room (approximately 750 × 480 cm). The ambient sound level in the room was 28 dBA, as measured with a microphone (Bruel & Kjaer, Type 4192) and an amplifier (Bruel & Kjaer, Type 2610). The apparatus that was used to assess participants’ echolocation ability was a size-discrimination task which was based on the apparatus used by Teng and Whitney (2011) and Thaler et al. (2014b). This apparatus is illustrated in Fig. 1. The apparatus consisted of a vertical metal frame with two horizontal metal bars. These bars were spaced 27.5 cm apart. Flat, circular discs that were made from acrylic (0.5 cm thick) and painted with primer could be placed at the end of these bars. The diameter of the largest disc (the reference disc) was 25.4 cm. The diameters of the five comparison discs were 5.1, 9, 13.5, 17.5, and 22.9 cm, respectively. Angular size differences were approximately 22.1°, 18.2°, 13.4°, 9°, and 2.9°, respectively. In total, each participant completed a total of 200 trials, 40 for each comparison disc. The placement of discs was randomized such that each comparison disc appeared equally often on the top and bottom location. A small speaker (JBL Micro II, 150–20.000 Hz) was attached to the forehead of the participant. The speaker was placed in the middle of the forehead with the lower part of the speaker just above the eyebrows. The speaker generated a 10 ms white noise signal (80 dB). We used a speaker to ensure the consistency of the sound signal that was being used. When the participant pressed a button, the sound signal was emitted. The experiment was performed using Presentation software (Version 17.1).

Participants performed the echolocation task in line with the procedure of Teng and Whitney (2011) and Thaler et al.(2014b). Participants were blindfolded and sat at a distance of 320 cm from the end wall of the room. The frame was placed at a distance of 50 cm measured from the discs to the participant’s ear. We opted for a larger distance than the distance (33 cm) used by Teng and Whitney (2011) and Thaler et al. (2014a), because our pilot studies showed a ceiling effect in performance at 33 cm. As a consequence, performance was high for all participants, showing almost no variance within the group, and excluding learning to occur. The height of the frame was adjusted in such a way that the participant’s ear was halfway between the two bars. The speaker was attached to the participants’ forehead (centre), and the button was placed in the hand of the participant. The participant completed two practice trials, which were followed by 50 test trials. After a short break of 5 min, another 50 trials followed. Between the trials, participants pressed their ears with their fingers to prevent them from hearing the experimenter placing the reference disc (i.e. the larger disc) on either the top or bottom bar and one of the five comparison discs on the remaining bar. In addition, even if a trial contained the same disc set-up as the trial before, the experimenter took the discs of the bar, and placed them back. This way, possible hints from noise from placing the discs or the amount of time it took the experimenter to place the discs, would be uninformative. Once the discs were placed, the experimenter tapped the participant on the shoulder to indicate that they could unblock their ears. First, the participant was asked to indicate whether they thought that the reference disc was on the top or bottom bar, with refraining to use sound (‘no-sound judgment’). By obtaining a no-sound judgment, a level of baseline performance or possible effects of background noise were established. This no-sound judgment might take into account any information from ambient noise or noise that arose from placing the discs. After the no-sound judgment, participants pressed the button to emit the sound signal from the speaker. Participants were allowed to produce sounds for up to 20 s to determine whether the reference disc was on the top or bottom bar (‘sound judgment’). The amount of emitted sound signals per trial and the time spent to make a judgment were registered. After each sound judgment, we registered the level of confidence of the participant concerning that sound judgment. Participants indicated how certain they were of their judgment on a scale of 1 (very uncertain) to 5 (very certain). Because we are interested in learning of echolocation, participants received correct/incorrect feedback after every trial (Herzog and Fahle 1997), in contrast to Teng and Whitney (2011) and Thaler et al. (2014a).

#### Spatial abilities

**Fig. 2:**
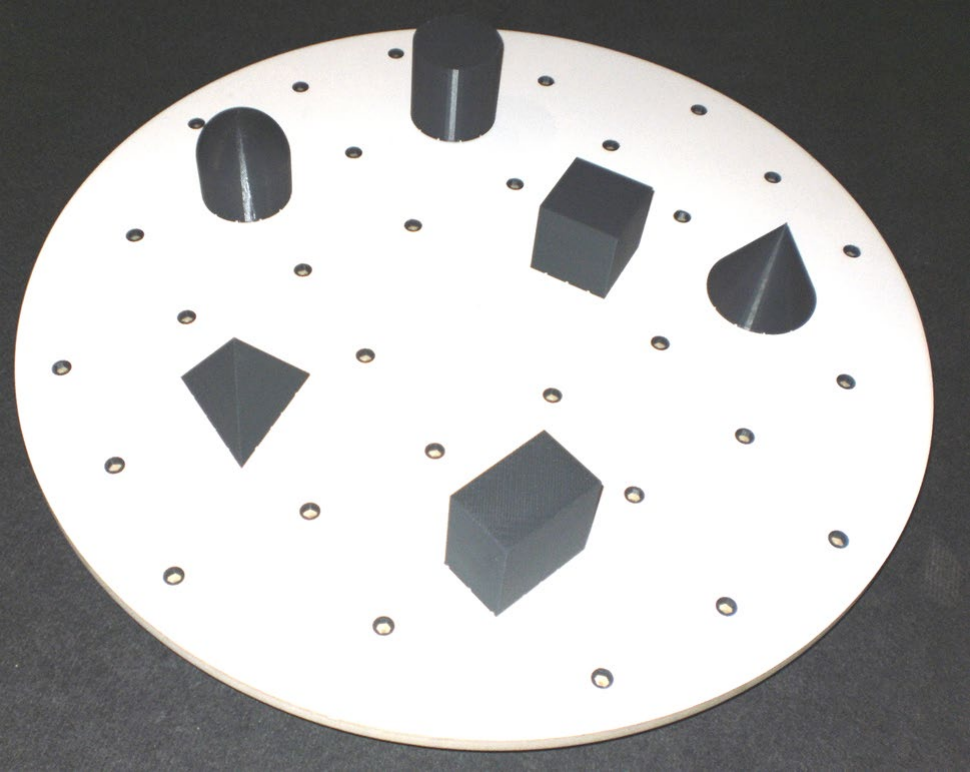
Set-up of the spatial abilities task

To measure participants’ spatial abilities in a non-visual manner, we used the following set-up, which was an adaptation of the set-up used by Simons and Wang (1998) and Pasqualotto and Newell (2007). A rotatable circular platform (70 cm in diameter) which contained 36 sunken position markers was placed on a table. In these markers, 6 objects (rectangular solid, cube, cylinder, sphere, cone, and pyramid) could be placed (see Fig. 2). The objects were placed on the platform at a random location, with the only constraint that the objects would have a minimal distance of 10 cm to each other. Subjects were blindfolded and sat in front of a table on which the platform was placed. In each trial, participants were given 60 s to learn where the objects were placed via haptic exploration. Subsequently, the experimenter randomly removed one or more objects from the platform and turned the platform 90° clockwise. Next, the participant placed the object(s) back on the (rotated) platform without time restriction. The number of objects that was removed from the platform increased every two trials, starting with one object in the first two trials, then two objects in the next two trials, etcetera. The possible total number of test trials was twelve. Participants completed three practice trials with feedback, followed by the test trials without feedback. The object was judged as correct if it was placed in the exact location or the location adjacent to that one. The test ended if the subject failed placing all objects correctly on the platform in two consecutive test trials with the same number of objects removed. The total score consisted of the number of correct trials with the maximum possible score of 12.

#### Working memory

To assess working memory, we used the digit span subtest of the Dutch Wechsler Adult Intelligence Scale (WAIS-IV-NL; Wechsler 2012). In this task, participants were asked to repeat a string of digits in the same order (forward recall), reversed order (backward recall), or ascending order (sort recall). These digits were presented verbally by the experimenter. The number of digits in a string increased every two trials. The test ended if the participant incorrectly repeated the string of digits with the same length twice. The total score consisted of the total number of correct series for the three tests combined. The total score was converted into a standard score based on Dutch norms. This task has good test–retest reliability, with a correlation coefficient of .88 (Wechsler 2012).

#### Sustained and divided attention

Sustained and divided attentions were measured using the Dutch version of the Paced Auditory Serial Addition Task (PASAT; Aarnoudse et al. 1995; Gronwall 1977). In this task, 60 single-digit numbers were presented to the participant. Participants were asked to add each digit to the one immediately preceding it and to immediately give the sum of these two digits. The test consisted of five blocks of 60 numbers with decreasing intervals between digits, ranging from 3.2 to 1.6 s. One total score was calculated, which consisted of the total number of correct additions, calculated over five blocks. The test–retest reliability of this task is good, with a correlation coefficient of .82 (Aarnoudse et al. 1995).

### Data analysis

The dependent variable was the percentage of correct judgments, which was calculated separately for each of the four sessions (one, two, three, and four), signal (no-sound and sound), and for each of the five angular size differences between the reference disc and the comparison disc (2.9°, 9°, 13.4°, 18.2°, and 22.1°). Chance performance was 50%. A three-way repeated measures ANOVA with within-subject factors session, signal, and angular size difference was computed. If the variances were not homogeneous (as determined with Mauchly’s test), the Greenhouse-Geisser correction was used. Furthermore, correlation analysis was conducted using correlation coefficients to determine the relationship between, on the one hand, the increase in echolocation ability and, on the other hand, the cognitive measures. We calculated the increase in echolocation ability as the difference between the sound and no-sound performance, looking at the difference in performance between the last and first session, across the angular size differences. An alpha level of .05 was considered statistically significant.

## Results

### Echolocation

**Fig. 3:**
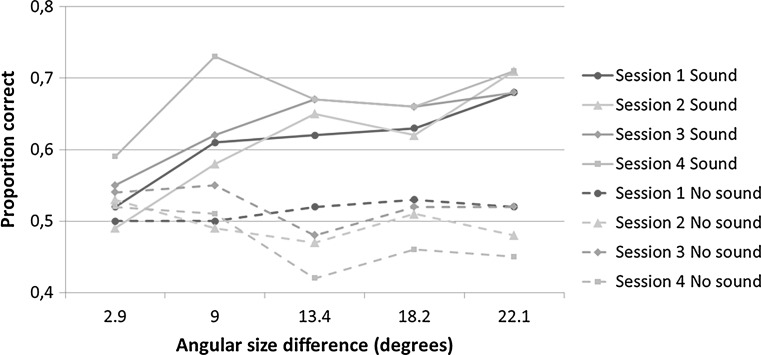
Proportion correct trials in the echolocation task for angular size difference, session, and signal condition, separately

Percentages correct trials in the sound condition of the echolocation task varied from 42 to 80% in the first session and from 48 to 96% in the last session. In line with the procedure of Teng and Whitney (2011) and Thaler et al. (2014), we computed a three-way repeated measures ANOVA with within-subject factors ‘session’ (one, two, three, and four), ‘signal’ (no-sound and sound), and ‘angular size difference’, i.e. the angular size difference between reference and comparison disc (2.9, 9, 13.4, 18.2, and 22.1). We found a significant main effect of signal [*F*(1, 17) = 14.354, *p* = .001, *η*_*p*_^2^ = .458]. Also, a main effect of angular size difference was found [*F*(4, 68) = 3.181, *p* = .019, *η*_*p*_^2^ = .158]. Furthermore, the analysis revealed a significant signal × session interaction [*F*(3, 51) = 4.432, *p* = .008, *η*_*p*_^2^ = .207] and a significant signal × angular size difference interaction [*F*(4, 68) = 8.182, *p* < .001, *η*_*p*_^2^ = .325]. No other effects were significant. Figure 3 shows performance (proportion correct judgments) in the echolocation task.

**Fig. 4:**
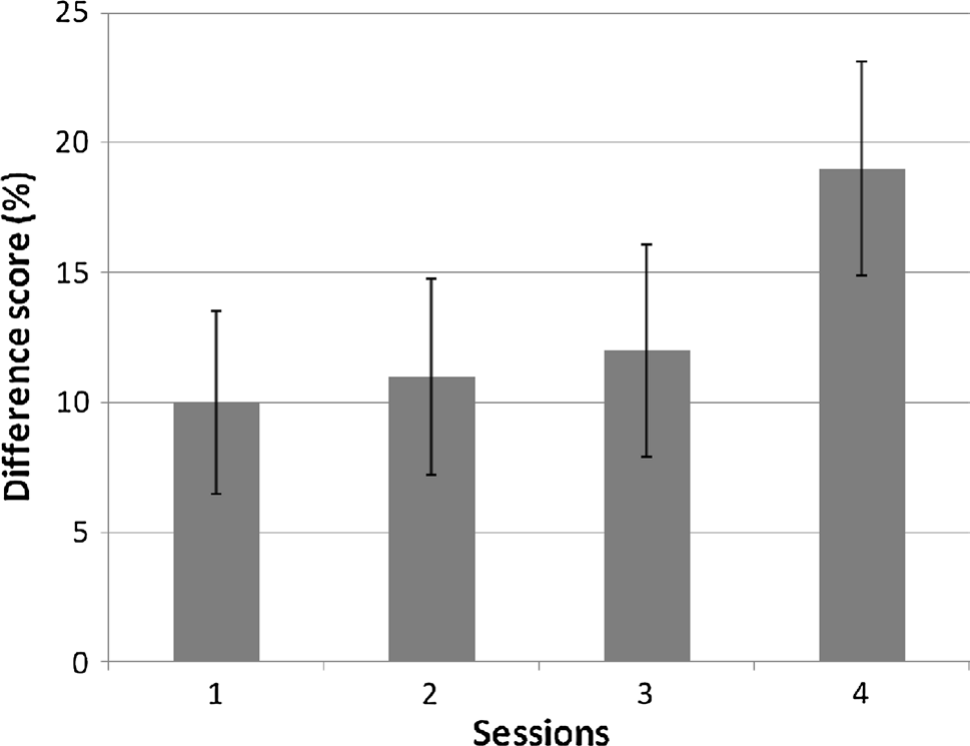
Difference scores (sound minus no-sound) in the echolocation task across sessions. *Error bars* represent SEM

Following up on the significant interaction between signal and session in the three-way (4 × 2 × 5) repeated measures ANOVA, we conducted a repeated measures ANOVA for sound and no-sound separately with session as the within factor. In the sound condition, we found a trend for a main effect of session [*F*(3, 51) = 2.315, *p* = .087, *η*_*p*_^2^ = .120]. Visual inspection of the sound condition suggested that the biggest difference was present between the first and last session. A paired *t* test performed on the first and last session reveals a significant difference [*t*(17) = −2.73, *p* = .023]. In the no-sound condition, there also was a trend for a main effect of session [*F*(3, 51) = 2.724, *p* = .054, *η*_*p*_^2^ = .138]. However, performance in the no-sound condition remained at chance level in every session (*p*_bonf_ > .05 for all sessions). Paired *t* tests revealed that in the first and third session, the difference between the no-sound and the sound condition just failed to reach conventional levels of statistical significance (*p*_bonf_ = .052). In the second and fourth session, there was a significant difference between the no-sound and the sound condition (*p*_bonf_ = .028 for the second and *p*_bonf_ < .004 for the last session). The difference scores between the sound and no-sound condition across sessions are shown in Fig. 4.

**Fig. 5:**
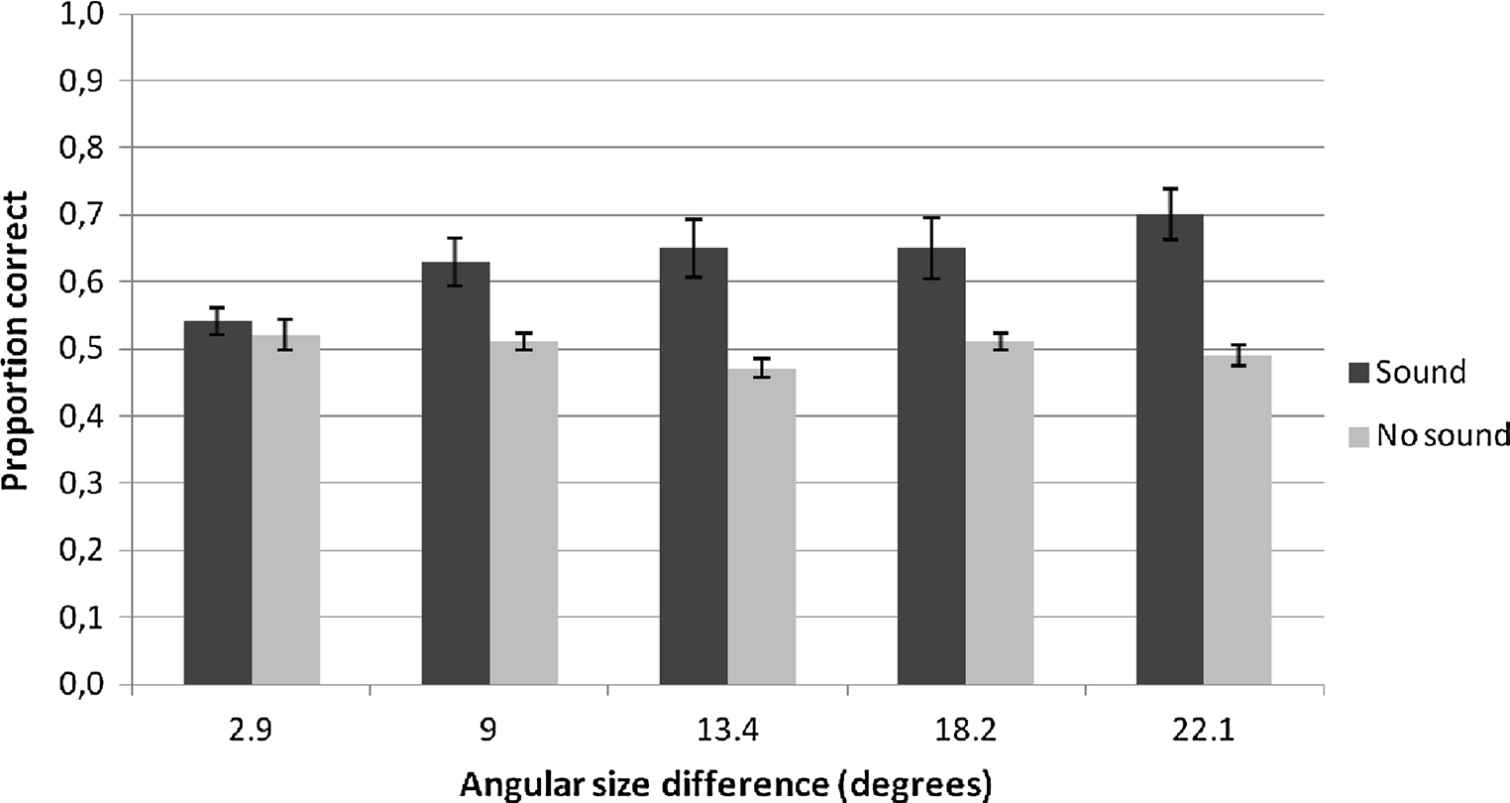
Proportion correct trials in the echolocation task for angular size difference and signal condition, separately. *Error bars* represent SEM

To further investigate the significant signal x angular size difference interaction found in the three-way (4 × 2 × 5) repeated measures ANOVA, we conducted a repeated measures ANOVA for the no-sound and the sound condition separately with angular size difference as within-subject factor. In the sound condition, we found a significant main effect of angular size difference [*F*(4, 68) = 8.099, *p* < .001, *η*_*p*_^2^ = .323], while in the no-sound condition, there was no significant main effect of angular size difference [*F*(4, 68) = 1.459, *p* = .22]. Furthermore, multiple *t* tests revealed that performance in the no-sound condition remained at chance level for every angular size difference (*p*_bonf_ > .05 for all sessions). Paired *t* tests performed on the sound and no-sound scores for every angular difference showed that only for the smallest difference (2.9°), participants did not perform better in the sound condition, compared to the no-sound condition (*p*_bonf_ > .05). For all other angular differences, participants performed better in the sound condition than the no-sound condition (*p*_bonf_ < .05; see Fig. 5).

The average amount of emitted sound signals per second in the echolocation task was 2.70 sound signals per second. After each trial, participants indicated how certain they were with regard to their judgment when using sound, ranging from 1 (very uncertain) to 5 (very certain). Mean score per trial across sessions was 2.47 with a standard deviation of 0.85. A repeated measures ANOVA performed on the certainty scores with within-factors session and angular size difference revealed a trend for a main effect of angular size difference [*F*(1.77, 30.14) = 3.028, *p* = .069, *η*_*p*_^2^ = .151]. The main effect of session and the interaction effect with session were not significant.

### Association between the increase in echolocation ability and the cognitive tests

**Fig. 6:**
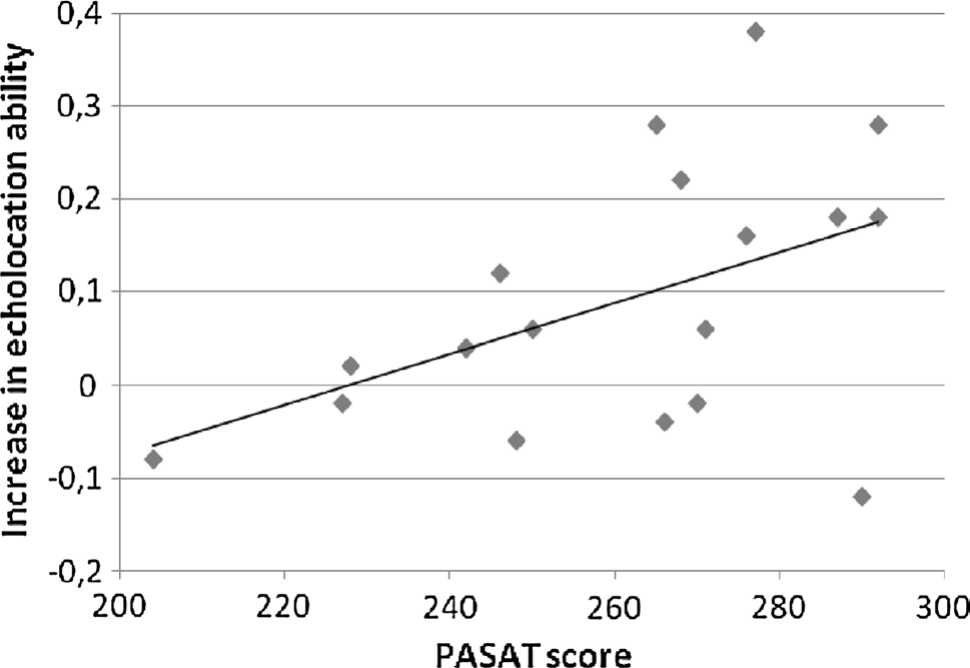
Participants’ PASAT score plotted against their increase in echolocation ability

Mean performance on the spatial cognition task was 5.22 correct trials with a standard deviation of 2.37. Mean digit span performance was 11.83 with a standard deviation of 3.43. Mean performance on the PASAT was 261.06 points with a standard deviation of 24.93. We used Pearson correlation coefficients because the data were normally distributed. The data revealed a significant positive correlation between the improvement in echolocation ability and performance on the PASAT [*r*(18) = .48, *p* = .042; see Fig. 6]. We additionally calculated Spearman’s rank correlation coefficient which revealed similar findings [*r*(18) = .46, *p* = .053]. Pearson correlations between the increase in echolocation ability and the other cognitive tests were not significant. When looking at the average difference between the sound minus no-sound condition across sessions instead of looking at the increase in echolocation ability, none of the cognitive tests showed a significant correlation with the average echolocation ability. Digit span correlated positively with performance on the PASAT [*r*(18) = .59, *p* = .011]; other correlations between the cognitive tests were not significant.

## Discussion

The purpose of this study was to examine the relationship between the learning of echolocation and individual factors that may affect echolocation learning: spatial cognition, working memory, and sustained and divided attention. In the echolocation task, when the participants did not produce a sound signal, that is, in the control condition, performance remained at chance level in every session. In the sound condition, there was a trend for a main effect of session on correct responses, but with a significant difference between the first and the last session. In addition to this, we found a significant main effect of angular size difference in the sound condition, but not in the no-sound condition. Our findings reveal that the improvement in echolocation ability is positively correlated with performance on the PASAT, while correlations between the improvement in echolocation ability and the other cognitive tests (spatial abilities and working memory) were not significant. We found no significant correlations between the average echolocation ability across sessions and the cognitive tests.

Previous studies also showed that it is possible for echo-naïve, sighted participants to learn an echolocation task. Teng and Whitney (2011) were the first to use this echolocation task and found that blindfolded sighted participants were able to make size-discrimination judgments. Thaler et al. (2014) recently replicated these experimental results. The absolute values of correct answers in our study are in very close agreement with those of previous studies: Average performance in the sound conditions in our study was 63.2%, in comparison with 62.3% (Thaler et al. 2014), and 70% (Teng and Whitney 2011). We also found that performance in the first session differed significantly from performance in the last session. As can be seen in Fig. 4, performance in the first three sessions had a rather modest increase whereas the largest rise occurred in session four. This indicates that a rise in performance level is present, but it does not occur early in the learning process. As mentioned, we additionally did find a positive correlation between the results on the PASAT and the ability to learn echolocation. The PASAT is a test of sustained and divided attention with good psychometric properties such as high levels of internal consistency and test–retest reliability. Obviously, the results do not point to a causal relation between the measured attentional abilities and the ability to learn echolocation. Even though it is important to further investigate the relationship, this finding could have implications for the understanding of learning echolocation. The positive correlation that was found provides leads for further research and for strategies in actual echolocation training.

A possible reason for the lack of a correlation with the performance on our spatial ability task might be that the measured spatial abilities are less relevant in the applied echolocation task. Additional research is warranted to further study the influence of specific spatial abilities preferably in a ‘real life’ setting (e.g. allowing actual navigation). Likewise, we did not find a significant correlation between the improvement in echolocation and working memory. Results from previous studies suggested an influence of executive processes on sound localization (Merat and Groeger 2003), and given the similarities between sound localization and echolocation, we hypothesized that working memory would also be of influence in echolocation. However, the exact relationship between sound localization and echolocation is not yet clear. This was illustrated in research by Wallmeier et al. (2013), in which adding a second object or sound source differently affected performance in a sound localization condition in comparison with an echolocation condition. Furthermore, research performed by Thaler et al. (2014) showed that source motion sounds are processed differently from echo motion sounds in the brain. Therefore, it is likely that the role of working memory is different in echolocation compared to sound localization.

Several differences existed in the set-up of the current echolocation experiment and previous studies. One of these differences was that we used a speaker through which participants could emit a sound signal, instead of participants using their own tongue click. It has been shown that familiarity with the used sound signal is of importance for determining the distance to a sound source (Zahorik et al. 2005). In addition, recent research that compared the use of a loudspeaker to the use of mouth clicks found that sighted people did better with the loudspeaker (Thaler and Castillo-Serrano 2016). Therefore, for participants that were unfamiliar with echolocation, using a constant sound signal produced by a loudspeaker rather than a self-produced tongue click could have decreased the difficulty of the echolocation task. This is illustrated by the ceiling effect in our pilot study where we used a distance of 33 cm. Because we found this ceiling effect, we increased the distance to 50 cm in the actual experiment. This increase distance resulted in a decreased angular size differences from 4.3°–31.6° to 2.9°–22.1°, making the task more difficult. The four participants with the highest performance in the first session had between 75 and 80% correct judgments in the first session. Two of these participants remained at this level in the fourth session while the other two increased their performance to 92 and 94%, respectively. This indicates that even for participants who did very well in the first session there was enough room for improvement, suggesting that our analysis was not negatively affected by ceiling effects.

Another consequence of using a speaker was the possibility of emitting more sound signals per trial than would be possible with a natural aural sound. With this, more information about the surroundings is obtained, which in turn would result in a larger increase in echolocation ability. However, the amount of emitted sound signals per second did not correlate with the increase in echolocation ability [*r*(18) = −.19, *p* = .45].

In the present study, we did not instruct participants to use a specific strategy for the echolocation task. They were instructed to use sound to perform the task and were told that they were allowed to move their head to the left, right, up, and down during the trials, as long as they did not move the rest of their body and kept their back against the chair. As a consequence, there was variation in the strategies that participants used to perform the task. Specifically, five participants did not move their heads at all during the trials. Their performance on the echolocation task while using sound did not differ from chance level. These findings seem to imply that a successful echolocation strategy must involve head movement. Therefore, we excluded these five participants from further analyses. A few other participants that did move their heads, were having trouble ‘finding’ the discs, i.e. they were scanning too low or too high.

Unexpectedly, we also found a trend towards change in the sessions of the no-sound condition. In the fourth session, performance in the no-sound condition dropped. In previous research, no such effect in the no-sound condition was found (Teng and Whitney 2011; Thaler et al. 2014). A possible explanation for this finding might be that participants used a strategy that benefitted their performance in the sound condition, but compromised their performance in the no-sound condition. Even when the participants produce no sound, there will be an ambient sound level. It is possible that participants were using the sounds from this ambient sound level, but in a counterproductive manner. When performing the sound condition of the task, participants learn that the locations where they hear the largest reflection of sound corresponds to the larger object. However, this strategy could be counterproductive when used in the no-sound condition, where the ambient sound level is actually more blocked by a larger disc.

It should be noted that all participants in the present study were university students aged between 18 and 24 years. One of the main issues for future research is to determine whether the relationships that were found are present in more diverse age groups with different education levels. Performance on the PASAT, for example, is significantly correlated with age and intelligence (Brittain et al. 1991). In addition, it is important to determine whether the relationships that we found are also present in visually impaired people. Research has shown that visually impaired people perform differently on tasks that asses spatial cognition, working memory, and divided attention in comparison with sighted people (Collignon et al. 2006; Kujala et al. 1997; Pasqualotto and Proulx 2012; Pigeon and Marin-Lamellet 2015). Additionally, visually impaired people generally have better echolocation ability than sighted people (Kolarik et al. 2014). Therefore, it is important to further investigate the relationships between echolocation ability and these individual factors in people with visual impairment in future research. Our finding that participants did learn to echolocate in addition to the relation to the PASAT warrants further study into the predictive value of the PASAT and the predictive value of the performance on the first session. For clinical practice, these results could be used as a screening to include individuals into an echolocation training.

Finally, our echolocation task used the size-discrimination paradigm. Echolocation in the real world, however, does not only consist of size discrimination, but also determination of the location of an object, the distance to an object, and the integration of these aspects. A next step for future research is to establish if the correlation between the learning of echolocation and attention also is present in other, real life, echolocation tasks.

## Conclusion

In this experimental study, we showed that sighted, blindfolded people are able to learn echolocation. Furthermore, we found a relationship between sustained and divided attention and the learning of echolocation. No such relationship was found for spatial abilities and working memory. These findings are a first step to advance our insights into the possible cognitive processes for learning to echolocate and might be implemented into intervention programs to train echolocation.
